# Measuring quality of life of adults with intellectual disabilities: Psychometric evaluation of the personal outcomes scale in the United Kingdom

**DOI:** 10.1111/jar.13189

**Published:** 2024-01-02

**Authors:** Helen Buxton, Manuel Gomes, Rafael Gafoor, Zac Taylor, Vaso Totsika

**Affiliations:** ^1^ Division of Psychiatry University College London London UK; ^2^ Department of Applied Health Research University College London London UK; ^3^ Comprehensive Clinical Trials Unit University College London London UK; ^4^ Royal Mencap Society London UK; ^5^ Centre for Research in Intellectual and Developmental Disabilities University of Warwick Coventry UK; ^6^ The Tavistock and Portman NHS Foundation Trust London UK; ^7^ MICARE Santiago Chile

**Keywords:** factor analysis, intellectual disability, psychometric testing, quality of life

## Abstract

**Background:**

The Personal Outcomes Scale (POS) is a scale developed to measure quality of life of adults (18+) with intellectual disability. Previous studies have reported good fit for Spanish and Portuguese language versions of POS.

**Aims:**

This study aimed to evaluate the factor structure of the English language version of POS when used to measure the quality of life of adults (18+) with intellectual disability in the UK.

**Materials and Methods:**

Analysis was conducted on POS data from 310 adults with an intellectual disability. First and second order factor models and multi‐level models were used to assess fit.

**Results:**

There was poor fit to the data for all tested models. We estimated that 23% of variance in POS scores was accounted for by interviewer cluster.

**Discussion:**

This was the first UK‐based evaluation of POS and our data did not confirm the factor structure of the POS measure. The identification of systematic variability within the dataset indicates that inter‐rater reliability is a potential limitation of the POS tool.

**Conclusion:**

Further research is needed to investigate inter‐rater reliability of POS interviewers and to explore factor structure.

## 1 INTRODUCTION

Having an intellectual disability is associated with an increased risk of physical health conditions such as epilepsy, diabetes and asthma (Liao et al., 2021), mental health conditions such as depression or anxiety (Cooper et al., 2015) and increased experiences of isolation and loneliness (Merrells et al., 2018); conditions which are associated with poorer Quality of Life (QoL) outcomes. Supporting people to live fulfilling lives is often cited as the goal of support provision and the measurement of QoL could provide a mechanism by which to monitor the quality of support services (Lombardi et al., 2019). However, there is a lack of standardised measures of QoL in routine use and this limits comparability of approaches (Chowdhury & Benson, 2011; McCarron et al., 2019).

One measurement tool designed to measure QoL of people with intellectual disability is the Personal Outcome Scale (POS). POS is based on a model of QoL developed by Schalock and Verdugo (Schalock et al., 2010). The model was generated based on input from focus groups, personal interviews and published literature and has been validated in cross cultural studies involving approximately 2000 participants (Jenaro et al., 2005; Schalock et al., 2005; Wang et al., 2010). The model positions QoL as relational—it is not as crude as the tally of things a person possesses or has achieved (i.e., their position in life), but it is the relationship between this position and the person's expectation of what they should have (Cummins, 2005; Welham et al., 2001). This example illustrates why the measurement of QoL must take account of both objective criteria of a person's life as well as subjective criteria – how a person feels about their life (Cummins, 2005). The Schalock and Verdugo model considers QoL as multi‐dimensional – comprised of eight core domains, all of which must be taken into consideration to understand a person's QoL (Schalock et al., 2010). The domains are personal development, self‐determination, interpersonal relationships, social inclusion, rights, and emotional, physical, and material well‐being. There is some evidence that the eight domains of QoL in this model can be aggregated into three higher order domains: independence (personal development and self‐determination), social participation (interpersonal relationships, social inclusion and rights) and wellbeing (physical, mental and material wellbeing), and that this hierarchical structure may be more universally applicable across different cultural contexts than the 8‐domain model (Wang et al., 2010).

POS was developed in 2008 in Belgium through consultation and pilot testing with people with intellectual disability, family members, direct support staff and experts (van Loon et al., 2010). Unlike some other scales (e.g., The Personal Wellbeing Index (Cummins et al., 2010); POS is designed to ensure inclusion of all people regardless of severity of communication and capacity needs. To achieve this, first interviewers are trained to use a semi‐structured approach to administration, which enables interviewers to adjust language and context to the participant's capacity needs (Claes et al., 2008). Second, a proxy version of the tool was developed to enable collection of response from staff or family member where participants do not have capacity to self‐report. Studies investigating inter‐respondent reliability between self‐report and proxy report have demonstrated moderate to high correlation (0.42–0.82) across the eight QoL domains, with subjective well‐being consistently demonstrating the weakest correlation across studies. Proxies tend to score participants' QoL lower than participants would themselves (Carbo‐Carrete et al., 2015; Claes et al., 2012; Simoes et al., 2015). These findings are aligned with previous research across a range of QoL measurement scales, especially in subjective domains (Cummins, 2002; Perry & Felce, 2002).

A POS manual and training process were also developed which stipulated that POS interviewers be trained over 2 days to ensure standardised interpretation of items and administration (Claes et al., 2008). A biproduct of the semi‐structured approach is the generation of substantial qualitative data for each participant, which organisations can use to inform individualised support strategies (van Loon et al., 2010). The measure is currently in use in research and practice in 12 countries: the majority in Europe, but also including Taiwan, Japan, and USA (S. Santos, personal communication 07 Nov 2021).

Although POS authors conducted research into the psychometric properties of POS at the time of development, they did not conduct factor analysis (Claes et al., 2008). Factor analysis is a statistical technique to evaluate how well observed data support the conceptual model a scale proposes to measure (Brown, 2015). Factor analysis assessment conducted in Spain (*N* = 529) demonstrated strong support for an eight‐factor structure and adequate support for a hierarchical 8 + 3 factor structure (Carbo‐Carrete et al., 2015). Another analysis with a larger sample (*n* = 1264) conducted in Portugal (Simoes et al., 2015) found that eight of the 48 items from the POS failed to load adequately and were therefore dropped from analysis on the basis that the removed items did not adequately discriminate from those retained.

When analysis was repeated with the remaining 40 items both the eight‐domain model and the hierarchical 8 + 3 factor structure were found to have adequate fit as estimated through small Root Mean Square Error of Approximation (RMSEA ≤ 0.55) and large Normed Fit Indices (NFI ≥ 0.97) and Relative Fit Indices (RFI ≥ 0.95) (Simoes et al., 2015). However, the eight‐domain model showed higher absolute and incremental goodness‐of‐fit indices, compared to the hierarchical model. (Simoes et al., 2015). Statistics indicating good fit are defined as RMSEA < 0.05; CFI > 0.9; TLI > 0.9 (Brown, 2015). Both these studies have confirmed that the factor structure of POS is the same regardless of the informant (Carbo‐Carrete et al., 2015; Simoes et al., 2016), which supports combining self‐report and proxy data for the purposes of future factor analyses.

The factor structure of POS as used in the English language has yet to be established. The differences in factor analysis findings for POS scales in Portuguese and Spanish versions demonstrate the importance of conducting factor analyses when POS is used in new settings. These studies have also not investigated the evidence for reporting POS scores as one global QoL score across all the domains or items (e.g., either a first order single factor, or a second‐order factor resultant from the eight first order factors).

The present study aimed to address these two gaps in the evidence, drawing on existing POS data collected as part of regular service provision by the UK charity Mencap (http://www.mencap.org.uk). The first objective of this study was to test the factor structure of the 48‐item POS with data from people with intellectual disability living in the UK. We also intended to replicate the factor analyses conducted in Portugal with a reduced version of POS (40‐items) (Simoes et al., 2016) to examine whether this would provide a better fit. All configurations were tested by combining data from self‐report and proxy report responses.

## 2 METHODS

### 2.1 Design

POS data were collected during interviews with adults recognised by adult social care services as having an intellectual disability; and were conducted as part of routine service provision. The sample is therefore comprised of people who were selected according to service provider convenience. Demographic data were made available from service registers. A total of 350 interviews were conducted between Aug 2018 and Dec 2022 inclusive. Of these, 250 were self‐report interviews and 100 were proxy report. Of the 350 interviews, 35 were repeat interviews (on the same person) and for five the proxy respondent was a family member as opposed to a staff member. These 40 interviews were excluded from analysis and thus the POS data set is comprised of 310 unique participants.

### 2.2 Participants

All participants were adults (18+) with intellectual disability for whom Mencap provides personal care and support. Participants lived either in supported living, residential care services or in their family home. Mencap involvement varied from a 24‐hr staff presence to staff who visit once or twice a week, as required by the participant's support needs. Mencap provided additional data to help describe participants. These included gender, age, geographic location, and date of interview.

There was a relatively large proportion of missing data relating to gender of participants (*n* = 95; 30.65%). Where gender was known there was a higher proportion of male participants (*n* = 121; 39.03%) than female (*n* = 94; 30.32%). The median age of participants was 53 years (Inter Quartile Range (IQR) 42–62 years) and participants were drawn from across England and Wales; the highest concentration (36.45%) being from around London, Southeast and East Anglia. Participant characteristics are displayed in Table 1.

**TABLE 1:** Participant demographic characteristics and interview administration (*n* = 310).

	*n*	(%)	POS score	
			Mean	SD
Age (years)				
(18–35)	22	(7.10)	112.32	(14.43)
(36–50)	95	(30.65)	114.32	(11.22)
(51–70)	129	(41.61)	110.71	(12.95)
(70+)	29	(9.35)	106.00	(11.70)
Missing	35	(11.29)	119.63	(8.05)
Gender				
Female	94	(30.32)	112.81	(11.94)
Male	121	(39.03)	110.68	(12.86)
Missing	95	(30.65)	114.51	(11.99)
Location				
London and Southeast and East Anglia	113	(36.45)	112.62	(12.25)
Wales, Southwest, and Midlands	101	(32.58)	112.05	(12.37)
Yorkshire and North of England	65	(20.97)	109.06	(12.97)
Northern Ireland	28	(9.03)	121.21	(7.55)
Missing	3	(0.97)	116	(4.58)
Year of interview				
2018	86	(27.74)	112.73	(12.04)
2019	115	(37.10)	112.53	(11.67)
2020	77	(24.84)	112.06	(14.27)
2021	31	(10.00)	112.68	(11.61)
Missing	1	(0.32)	–	–
Interview conducted before start of COVID‐19 national lockdowns in the UK (March 2020)				
Post‐March 2020	34	(10.97)	113.21	(11.24)
Pre‐March 2020	275	(88.71)	112.39	(12.56)
Missing	1	(0.32)	–	–
Type of report				
Self‐report	236	(76.13)	115.86	(10.32)
Proxy report	74	(23.87)	101.78	(12.39)

As shown in Table 1, most interviews were conducted in the first 2 years of data collection (2018–2019: 64.84%), while 88.71% of interviews were conducted prior to the start of the UK COVID‐19 lockdowns. Just 3/4 of interviews were self‐report, and as found in previous studies mean proxy scores were about 15 points lower than those for self‐report scores (Carbo‐Carrete et al., 2015; Claes et al., 2012; Simoes et al., 2015).

### 2.3 Measures

The POS contains 48 items which are rated on a 3‐point Likert scale; though the response options are specific to each item (Claes et al., 2008). For example: “Do you talk to people who live or work near to you?” provides the response options: “Often,” “Sometimes,” “Rarely or never”; whereas “In general, how healthy do you feel?” provides response options: “Very healthy,” “Okay,” “Sick” or “Ill.” Items and response options for both self‐report and proxy versions are very similar and intended to measure the same constructs.

All interviews were conducted by members of Mencap staff who had completed standardised training. Staff self‐selected to be POS interviewers as a professional development opportunity. Training was delivered by Mencap staff who had qualified at POS ‘master trainer’ level and POS ‘co‐trainer’ level. Qualification to training roles is dependent on conducting at least 20 POS interviews and having been observed by a master trainer for three of these. Inter‐rater reliability was not estimated as part of the training process.

### 2.4 Analysis

#### 2.4.1 Confirmatory factor analysis of POS

The original eight factor 48‐item POS scale was tested using Confirmatory Factor Analysis (CFA). Alternative configurations were tested using the 48 items including 8 + 3 higher order domains: 8 + 1 higher order and one global QoL factor. A reduced 40‐item scale, based on findings published in a previous study (Simoes et al., 2016) was also tested using CFA.

Sample size is an important consideration for CFA, however the minimum sample required is inversely related to the model degrees of freedom *(d)* (MacCallum et al., 1996). All the models tested have large *d* due to the relatively large number of items in the POS scale (48), suggesting we can be confident that the sample is adequate to assess model fit. Factorability of the correlation matrix was further tested through Bartlett's test of sphericity and the Kaiser–Meyer–Olkin (KMO) test of sampling adequacy. Factor analysis is dependent on a *p*‐value ≥0.05 for Bartlett's test, and KMO values ≥0.08.

Model fit was assessed using the RMSEA and its 90% Confidence Interval (CI). Values ≤0.05 were considered close fit (Hu & Bentler, 1999). The Comparative Fit Index (CFI) and the Tucker Lewis fit Index (TLI) were calculated; with values ≥0.95 considered good fit (Hu & Bentler, 1999). The Standardised Root Mean Square Residual (SRMR) was also considered, with values ≤0.08 considered good fit (Brown, 2015). Chi‐square test results are also reported to assess the fit between the hypothesised model and the data – where the null hypothesis represents perfect fit.

#### 2.4.2 Understanding reasons for observed fit

Descriptive statistics, including response proportions for each item and inter‐item correlations were generated and inspected for evidence of problematic items (Sexton et al., 2013). As data are ordinal, polychoric correlations were calculated. To extract components from the data measured variables must be sufficiently intercorrelated, and the majority of correlations should exceed 0.3 (Hair et al., 2014).

Due to the semi‐structured nature of administration of POS, and the large number of interviewers involved in data collection, we hypothesised that a large proportion of variance in the data set was influenced by between‐interviewer variability. There are a number of ways interviewers could have introduced error into the data—this could be at participant selection, for example, in the way that they made contact and gained cooperation from participants, or it could be measurement error introduced in the way that interviewers deliver questions (West & Blom, 2017).

To explore interviewer‐specific effect Multi‐Level Models (MLM) with interviewer ID as random intercepts were conducted. Based on complete case analysis 36 interviewers conducted a median of 2 interviews each (IQR 1–3). However, the number of interviews ranged from 1 to 26. Only four interviewers conducted more than 10 interviews.

MLM allow the variance to be partitioned into two levels—POS scores were regressed on demographics (age, gender, and geographic location) and interview administration (year and type of interview) while random intercepts at level 2 (interviewer ID) allowed for clustering at that level. We initially estimated an unadjusted model and computed the Intra‐class Correlation coefficient (ICC)—the proportion of variability in the total QoL score that exists between interviewers. Participant level demographic variables (age, gender, and geographic region) were then added, followed by process variables (year of interview, pre/post start of Covid‐19 lockdowns, self, or proxy report) to explore if the interviewer effect was attenuated by any of these variables. Model significance was tested through the serial likelihood ratio test, which if statistically significant (*p* < 0.05), indicated that the covariate‐adjusted model is a better fit to the data than the unadjusted model (where only the interviewer‐specific random effect was included).

#### 2.4.3 Missing data

**FIGURE 1:**
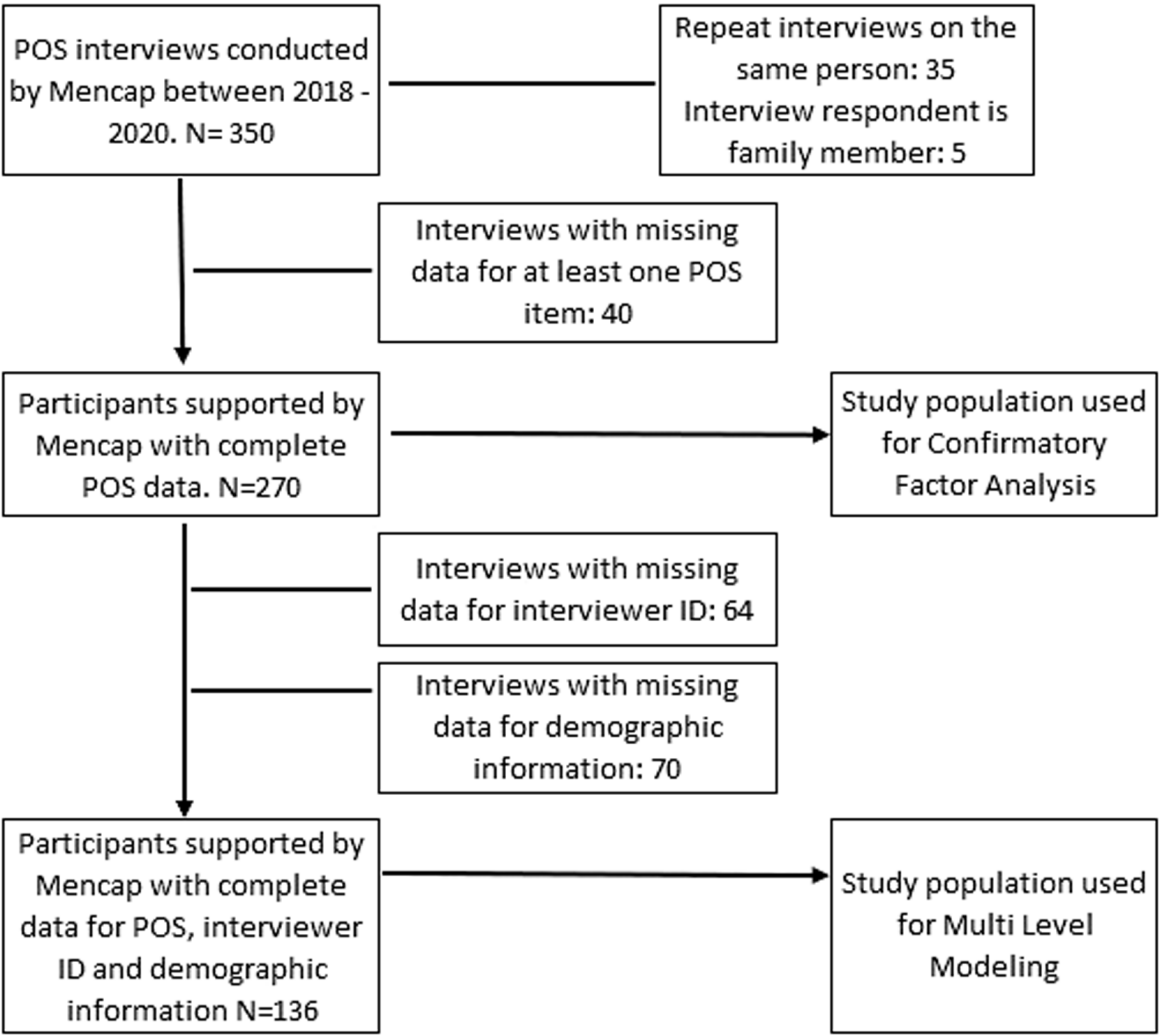
STrengthening the Reporting of OBservational studies in Epidemiology diagram: Illustrating selection of samples used for study analyses.

The STrengthening the Reporting of OBservational studies in Epidemiology (STROBE) diagram illustrates the patient selection process in Figure 1. There were 310 unique interviews available for analysis—236 interviews were self‐report and 74 were proxy report. Two hundred and seventy of the interviews (87.10%) had complete data for all POS items. The greatest amount of missing data per participant was nine items but this was for only one participant. Data is missing for items if the participant chose not to answer a specific question, the answer is unknown or due to input error. As the proportion of observations with missing data was relatively small and visual inspection revealed no systematic missing data pattern or predictors of missingness, complete case analysis (n = 270) was conducted for CFA.

MLM analysis required data on interviewer ID and participant demographics. Sixty‐four interviews were missing data on interviewer ID, all of which were conducted prior to February 2020 when Mencap was using a less robust form of data capture (MS Forms). Data are missing through user error (leaving field blank, entering their name inconsistently e.g., just first name or initials). Because interviewer ID was used to define the higher‐level group in MLM, those interviews with data missing on interviewer ID were excluded from analysis. Data were taken from Mencap service registers to provide demographic information to participant observations. However, variables are not mandatory fields on Mencap's databases and inconsistent use resulted in missing data. A further 70 observations were missing data on at least one of the following variables: interviewer ID, gender, age, or geographic location. Multilevel analysis therefore comprises a reduced sample of 136 observations across 36 interviewers. This analysis assumed that, conditional on both individual characteristics and interviewer ID being present, the data were missing completely at random.

### 2.5 Implementation

MPlus (version 8) was used for all factor analyses (Muthén & Muthén, 2017). For CFA the Weighted Least‐Squares Mean and Variance‐adjusted (WLSMV) estimator was used to account for the categorical nature of the items (Bandalos, 2014; Muthén & Muthén, 2017). Stata v17 (StataCorp, 2019) was used to describe the data and for multi‐level modelling.

As the study was a secondary analysis of routinely collected service data, POS interview data and demographic information were linked by the data owners (Mencap) prior to sharing an anonymised database with the research team. Ethical approval for the current study was provided by UCL research ethics committee (Project ID: 19259/002).

## 3 RESULTS

### 3.1 Results of confirmatory factor analyses

Results from the CFA to test the theoretical QoL measurement models are displayed in Table 2. Fit indices indicated poor fit to the data for all possible factor structures of the 48‐item scale. Items were then reduced to 40 to replicate previous CFA from Portugal (Simoes et al., 2016). However, fit did not improve with the 40‐item POS data.

**TABLE 2:** Fit statistics for POS (*n* = 270).

Model	Chi square (p)	CFI	TLI	RMSEA (90% CI)	SRMR
8 factor, 48 items	1967.13 (<0.001)	0.750	0.732	0.057 (0.053–0.060)	0.121
8 + 3 factor 48 items	2088.96 (<0.001)	0.722	0.706	0.059 (0.056–0.063)	0.127
8 + 1 factor 48 items	2072.00 (<0.001)	0.727	0.713	0.059 (0.055–0.063)	0.128
1 factor 48 items	2362.26 (<0.001)	0.650	0.063	0.066 (0.063–0.070)	0.137
8 factor 40 items^a^	1357.56 (<0.001)	0.740	0.715	0.058 (0.053–0.063)	0.115

Abbreviations: CFI, Comparative Fit Index; POS, Personal Outcomes Scale; RMSEA, Root Mean Square Error of Approximation; SRMR, Standardised Root Mean Square Residual; TLI, Tucker Lewis fit Index.

^a^ Model includes 40 items retained following CFA with POS data in a previous study (Simoes et al., 2016).

### 3.2 Investigating observed model fit

#### 3.2.1 Suitability of data for factor analysis

Although statistical test results indicated that data were suitable for factor analysis (p value: Bartlett's test <0.001; KMO: 0.812), examination of the polychoric correlation matrix for POS data found insufficient intercorrelations. Only 44.22% of all possible between item correlations reached the minimum expected cut off 0.3, and a further 30.02% of possible values returned negative correlations.

#### 3.2.2 Factors accounting for variance in POS scores

Examination of correlation matrices by interviewer indicated that there was variance in between‐item correlation dependent on person who had conducted the interview.

**FIGURE 2:**
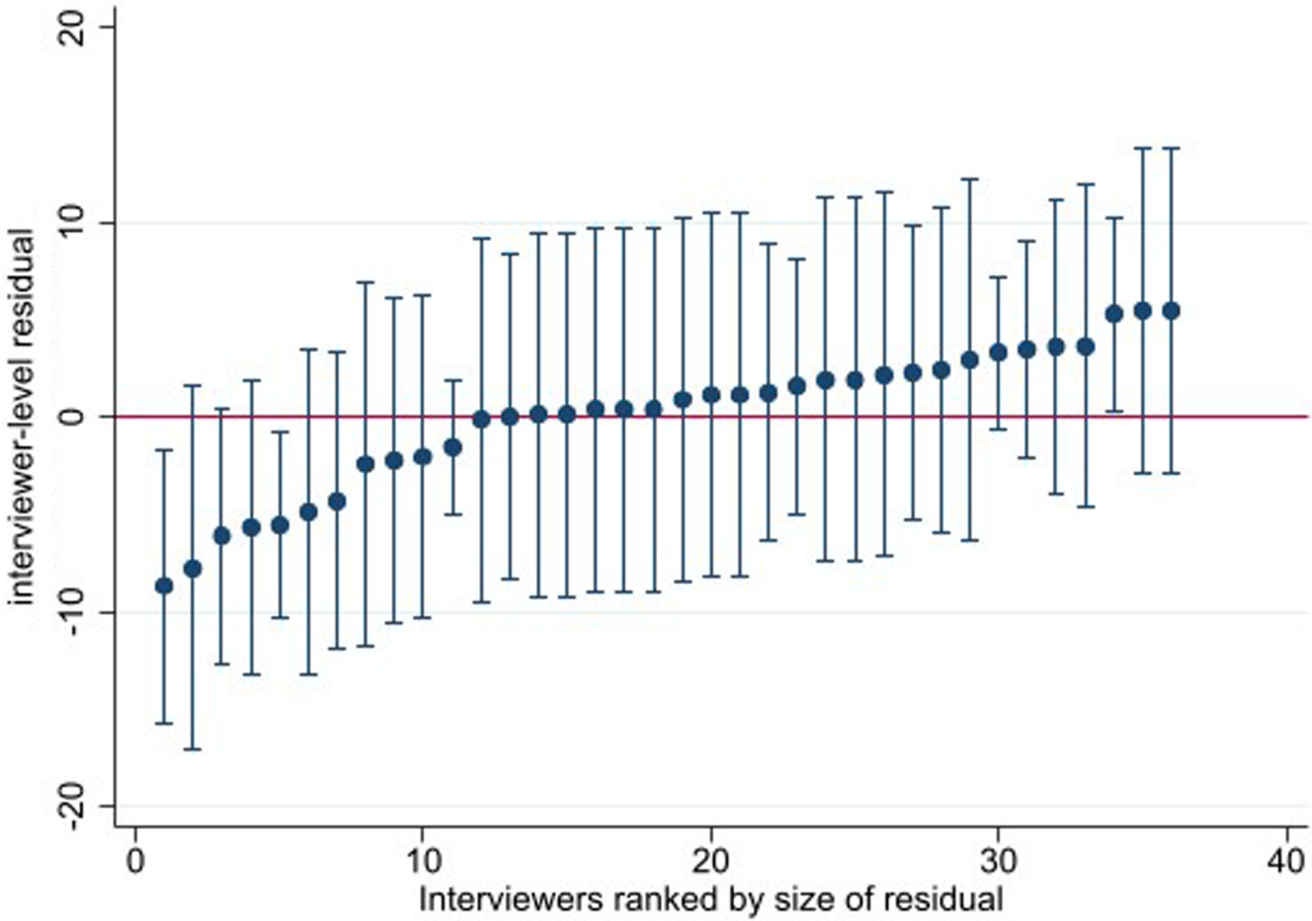
Caterpillar plot: Visualising variation between interviewer (*n* = 136).

To explore this further we conducted analysis on only the data which was complete across all demographic and process variables. Predicted POS score for the 136 observations when clustered by the 36 interviewers was 113.51 (95% CI 110.76–116.25). The variability in random intercepts for interviewers is illustrated by caterpillar plot (Figure 2). The wide confidence intervals displayed are likely a result of small numbers of interviews per interviewer, nonetheless the plot depicts variation among interviewer cluster means. Unexplained interviewer level variance was calculated at 30.44 (95% CI 10.82–85.68) and unexplained residual variance at 88.93 (95% CI 67.83–116.60). The ICC statistic indicated that the proportion of total variance that is accounted for by clustering at the interviewer level was 25.50% (95% CI 0.099–0.516).

Models were rerun inclusive of participant characteristics and interview administration variables (see Table 3). None of the participant characteristics was found to be significantly associated with POS score, but type of report (self or proxy) did demonstrate association. The ICC coefficient for the model with type of report included at participant level further supports the finding that a large proportion of total variance in POS score is accounted for by interviewer cluster (ICC: 0.2383 95% CI 0.0817–0.5239).

**TABLE 3:** Summary comparison of multi‐level models (*n* = 136).

Terms	Interim model 1 (demographics)	Interim model 2 (process)	Final model
	coefficient (95% CI)	coefficient (95% CI)	coefficient (95% CI)
Fixed effects			
Average POS score	121.11 (113.99–128.23)	106.56 (100.89–112.23)	105.10 (101.27–108.93)
Gender			
Female	1		
Male	−3.46 (−6.91 to 0.15)		
Age	−0.09 (−0.21 to 0.03)		
Location			
London & S. E	1		
Wales, S.W. & Central	−2.87 (−7.72 to 1.98)		
North of England	−4.38 (−10.02 to 1.24)		
Year			
2018		1	
2019		−2.01 (−6.85 to 2.82)	
2020		0.32 (−5.8 to 6.44)	
2021		−2.48 (−8.75 to 3.79)	
Report type			
Proxy		1	1
Self‐report		10.06 (6.23–13.88)	10.51 (6.81–14.21)
Random effects			
Interviewer level—variance	25.72 (8.99–73.53)	22.22 (6.68–73.91)	22.90 (7.29–71.97)
Residual variance	86.16 (65.95–112.57)	72.59 (55.01–95.79)	73.21 (55.63–96.35)
Intraclass coefficient	0.230 (0.087–0.484)	0.234 (0.076–0.534)	0.238 (0.082–0.524)
Likelihood ratio test	chi2 = 6.37	chi2 = 29.31	
*p*‐Value	0.173	<0.001	<0.001

## 4 DISCUSSION

This study aimed to explore how well POS data from a sample of people with intellectual disability living in the UK fit the conceptual model of QoL which the scale is intended to measure. Fit indices indicated poor fit to the data for all tested models. Factor analyses of UK POS data did not support an 8‐domain QoL model, an 8 + 3, 1, or an 8 + 1 domain QoL model. These findings do not replicate previous evidence published from research groups in Spain and Portugal which demonstrated acceptable fit for both the 8‐domain QoL model and the 8 + 3 domain models (Carbo‐Carrete et al., 2015; Simoes et al., 2016). Potentially this could be explained by differences in participant characteristics. In both previous studies the majority of participants lived in their family home and had mild to moderate intellectual disabilities—in Portugal a criterion for inclusion was verbal capacity to answer the self‐report measure (Simoes et al., 2016). The majority of participants in our sample lived in supported accommodation and data were not available to categorise participants by intellectual disability level. We do know that the UK sample included people with severe intellectual disability, and verbal capacity was not considered a pre‐requisite for self‐report. Where appropriate, people were supported to self‐report through the engagement of communication aids and communication partners. UK data have therefore been collected from people with greater variability of severity of intellectual disability than those included in previous studies. Further analysis of the data collected in Spain examined the effect of severity of intellectual disability on individual item functioning and found that the scores of a significant number of items were affected by severity (Carbo‐Carrete et al., 2019). Authors suggest this indicates the need for broader discussion of the adequacy of definitions of QoL dimensions and indicators for the whole spectrum of people with intellectual disability. The poor fit found in our study may reflect in some part the question of adequacy of POS indicators when used to measure QoL of people with severe intellectual disability, but further research is required to explore this.

In our study the lack of a clear factor structure was explained mathematically by weak inter‐item correlation (Hair et al., 2014). We hypothesised that this variability in item correlation may have resulted from interviewer bias – systematic error introduced through the interviewer's gathering of selective data or their influence over the participant's response (Jager et al., 2020). Multi‐level models were fitted to examine the effect of interviewer and our results indicated that 23.83% of variance in POS score was accounted for by interviewer cluster.

Bias in the sample could have been introduced at selection as participants were selected according to organisation and interviewer convenience (West & Blom, 2017). (e.g., interviewers may have been more likely to approach participants at services where they already held a relationship with either staff or participants). In addition measurement error may have been introduced during the interview as interviewers are trained to apply context which is meaningful to the participant to items to aid understanding (Claes et al., 2008), and any slight variation in interviewer style or technique (e.g., being suggestive or hasty); in attitude of the interviewer; or in interviewer interpretation of items could introduce variability.

A review of the literature on interviewer effect (Schaeffer et al., 2010) found that survey questions which were attitudinal, sensitive, ambiguous, complex or open‐ended were more likely to introduce variable interviewer effects. POS questions are not open‐ended but some could be classed as sensitive (e.g., do you think you are important to your family?); ambiguous (e.g., Are you able to demonstrate the skills that you have and the things that you can do?) or complex (e.g., Could you have a partner if you wanted one?). These types of questions may provide more opportunity for probing or using a more conversational format to deliver questions (West & Blom, 2017). Although previous studies have shown that these techniques tend to have a positive effect on response quality (West & Blom, 2017) the dynamic between interviewer and interviewee may result in higher likelihood of acquiesce bias (e.g., through leading questions) or recall bias (e.g., through excessive probing) (Bergen & Labonté, 2020). In addition, previous studies have found that interviewer demographics such as gender, age and race are related to response quality (West & Blom, 2017). In our study data related to interviewer demographics is not available to examine this further.

Increased interview standardisation has been shown to reduce interviewer effect (Jager et al., 2020), however the flexibility of scale administration is a key property to recommend POS for use with people with intellectual disability. POS authors have specified that interviewers be trained to administer the scale as a conversation, sensitive to the communication needs and styles of the person being interviewed, and ordering the questions aligned with the natural flow of conversation (Claes et al., 2008). A conventional measurement scale with fixed order and wording of items would result in the unnecessary exclusion of many people from self‐report due to varying communication and comprehension needs. Theoretically, QoL must include consideration of a person's subjective experience, which pragmatically means that wherever possible the person whose QoL is being measured should be the one to rate themselves against given criteria (Schalock et al., 2010). Therefore, efforts to increase standardisation of the scale itself to reduce interviewer bias could be counterproductive by introducing selection bias into the sample.

Measures which use a semi structured interview process to assess a quantitative metric have been shown to have good reliability (e.g., Ford et al., 2018; Lobbestael et al., 2011), therefore the flexibility allowed in POS administration should not automatically be assumed to be a challenge to reliability. However. to our knowledge no study has formally examined inter‐rater reliability of POS interviewer. POS data collection in both the Portuguese and Spanish studies used large numbers of interviewers and although interviewers were trained in POS administration, these studies do not report methods to ensure calibration of interviewers (Carbo‐Carrete et al., 2015; Simoes et al., 2016). We were not able to estimate inter‐rater reliability. However, the estimated size of the proportion of variance accounted for by interviewer clustering suggests that a significant proportion of the variability introduced into POS scores was attributable to the interviewer. Further research is needed to explore the inter‐rater reliability of the measure.

Anecdotally, flexibility in the administration results in high acceptability of the measure by support staff and participants (Mencap, personal communication, May 2022). Potentially, this flexibility is further enhanced by Mencap's employment of direct support staff as interviewers. Direct support staff are highly skilled and experienced in adjusting their communication style and tactic knowledge to the needs of the people they support (Barken & Armstrong, 2018). Authors note from observing POS interviews that interviewers clarify and avoid bias by applying context from previous questions to further explore a response. As a qualitative interview technique this demonstrates expertise (Lavee & Itzchakov, 2021), but for the purposes of extracting a quantitative score from the interview, it is dependent on interviewers sharing exact interpretation of each of the questions and response options. The questions used in the POS scale are intended to be broad enough to apply to multiple contexts, for example, the question “Are you learning to do new things?” could refer to formal courses undertaken at college, or to tasks around the home done with one‐to‐one support, such as chopping vegetables. Interviewers must share awareness of the broad applicability of these items, in order to prompt interviewees further. Any slight variation in interviewer interpretation could introduce extra variability into the measure, and this may explain some of the variation seen in the results of this study.

## 5 LIMITATIONS

This study analysed data collected in the context of regular service provision by a single provider. As such, participants were recruited according to convenience, and this could have introduced selection bias into the sample. In addition, as data were only collected by one provider, we cannot say how the inclusion of participants from different providers may have influenced results. Results may not be widely generalisable.

A high degree of missing data was observed for participant demographic information, while other important participant descriptors (e.g., verbal communication skills and level of intellectual disability severity) were not available. It is therefore not possible to understand how differences between respondents interact with the psychometric properties of POS. For example, the standard deviation in POS score is much lower among participants in Northern Ireland, but we cannot explore this difference further due to the scarcity of demographic information available. The large proportion of missing demographic data resulted in reductions in the sample size available as we restricted cases according to criteria of available demographic data. In addition, the sparsity of observations per cluster resulted in less precise estimates. Although our results were not able to demonstrate a statistically significant association between POS score and gender, age, or geographic location we would caution that this is likely the result of inadequate sample for the complexity of analysis.

Our results suggest that interviewer effect is compounding variability in the data already present due to the multi‐item nature of the questionnaire, but we cannot conclude that other factors, such as severity of intellectual disability are not also a source of score variability. Further research is required to explore these factors.

## 6 RECOMMENDATIONS

Our study findings highlight the need to establish better scoring consistency among interviewers prior to administration of the POS. During POS interviewer training, further emphasis should be placed on calibration between interviewers of interpretation of response options. Strong inter‐rater reliability may need to be established prior to interviewing people. Aligned with methodology used in a previous study (Ford et al., 2018), interviewers could be asked to watch and independently assess recorded demonstration interviews until all interviewers reach ≥80% agreement with expert ratings. Following calibration of interviewers formal assessment of inter‐rater reliability should be conducted through duplicate interviewing of the same participant (Gisev et al., 2013). The lack of studies assessing inter‐rater reliability across any of the existing POS literature may be due to not wishing to overburden participant time. However, our study highlights that the flexibility in administration of the scale may undermine the validity of the POS and warrants investigation.

## AUTHOR CONTRIBUTIONS

Vaso Totsika and Helen Buxton designed the study with contributions from Manuel Gomes and Rafael Gafoor. Helen Buxton cleaned and managed the data and performed formal data analysis with support from Vaso Totsika. Helen Buxton drafted the manuscript. Rafael Gafoor and Manuel Gomes provided oversight for the statistical analyses. All authors reviewed and edited the subsequent drafts and approved the final version of the manuscript.

## FUNDING INFORMATION

This study was co‐funded by the Economic Social Research Council and Royal Mencap Society as part of a ESRC Studentship at the UCL, Bloomsbury and East London Doctoral Training Partnership (UBEL DTP). The funders played no role in the study design, analysis, or interpretation of data or in the writing of the manuscript.

## CONFLICT OF INTEREST STATEMENT

The authors declare no conflict of interest.

## Supporting information


**Data S1:** Supporting Information.

## Data Availability

The data that support the findings of this study are available from the corresponding author upon reasonable request.

## References

[jar13189-bib-0001] Bandalos, D. L. (2014). Relative performance of categorical diagonally weighted least squares and robust maximum likelihood estimation. Structural Equation Modeling: A Multidisciplinary Journal, 21(1), 102–116. 10.1080/10705511.2014.859510

[jar13189-bib-0002] Barken, R. , & Armstrong, P. (2018). Skills of workers in long‐term residential care: Exploring complexities, challenges, and opportunities. Ageing International, 43(1), 110–122.

[jar13189-bib-0003] Bergen, N. , & Labonté, R. (2020). “Everything is perfect, and we have no problems”: Detecting and limiting social desirability bias in qualitative research. Qualitative Health Research, 30(5), 783–792. 31830860 10.1177/1049732319889354

[jar13189-bib-0004] Brown, T. A. (2015). Confirmatory factor analysis for applied research (2nd ed.). Guilford press.

[jar13189-bib-0005] Carbo‐Carrete, M. , Guardia‐Olmos, J. , & Gine, C. (2015). Psychometric properties of the Spanish version of the personal outcomes scale. International Journal of Clinical and Health Psychology, 15(3), 236–252. 10.1016/j.ijchp.2015.04.002 30487841 PMC6224865

[jar13189-bib-0006] Carbo‐Carrete, M. , Guardia‐Olmos, J. , Pero‐Cebollero, M. , & Gine, C. (2019). Impact of the intellectual disability severity in the Spanish personal outcomes scale. Journal of Intellectual Disability Research, 63(10), 1221–1233. 10.1111/jir.12634 31115999

[jar13189-bib-0007] Chowdhury, M. , & Benson, B. A. (2011). Deinstitutionalization and quality of life of individuals with intellectual disability: A review of the international literature. Journal of Policy and Practice in Intellectual Disabilities, 8(4), 256–265.

[jar13189-bib-0008] Claes, C. , van Loon, J. , Van Hove, G. , & Schalock, R. (2008). POS: Personal support outcomes scale. Manual. Retrieved from https://hcpbs.org/wp-content/uploads/2018/10/vanloonposmanual.pdf

[jar13189-bib-0009] Claes, C. , Vandevelde, S. , Van Hove, G. , van Loon, J. , Verschelden, G. , & Schalock, R. (2012). Relationship between self‐report and proxy ratings on assessed personal quality of life‐related outcomes. Journal of Policy and Practice in Intellectual Disabilities, 9(3), 159–165.

[jar13189-bib-0010] Cooper, S. A. , McLean, G. , Guthrie, B. , McConnachie, A. , Mercer, S. , Sullivan, F. , & Morrison, J. (2015). Multiple physical and mental health comorbidity in adults with intellectual disabilities: Population‐based cross‐sectional analysis. BMC Family Practice, 16(1), 110. 10.1186/s12875-015-0329-3 26310664 PMC4551707

[jar13189-bib-0011] Cummins, R. (2002). Proxy responding for subjective well‐being: A review. International Review of Research in Mental Retardation, 25, 183–207.

[jar13189-bib-0012] Cummins, R. A. (2005). Moving from the quality of life concept to a theory. Journal of Intellectual Disability Research, 49(Pt 10), 699–706. 10.1111/j.1365-2788.2005.00738.x 16162114

[jar13189-bib-0013] Cummins, R. A. , Lau, A. L. D. , Davey, G. , & McGillivray, J. (2010). Measuring subjective wellbeing: The personal wellbeing index – Intellectual disability. In R. Kober (Ed.), Enhancing the quality of life of people with intellectual disabilities. From theory to practice (pp. 33–46). Springer.

[jar13189-bib-0014] Ford, J. D. , Spinazzola, J. , van der Kolk, B. , & Grasso, D. J. (2018). Toward an empirically based developmental trauma disorder diagnosis for children: Factor structure, item characteristics, reliability, and validity of the developmental trauma disorder semi‐structured interview. The Journal of Clinical Psychiatry, 79(5), 4337. 10.4088/JCP.17m11675 30256549

[jar13189-bib-0015] Gisev, N. , Bell, J. S. , & Chen, T. F. (2013). Interrater agreement and interrater reliability: Key concepts, approaches, and applications. Research in Social & Administrative Pharmacy, 9(3), 330–338. 10.1016/j.sapharm.2012.04.004 22695215

[jar13189-bib-0016] Hair, J. F. , Black, W. , Babin, B. , & Anderson, R. (2014). Multivariate data analysis (Global Edition (7th ed)). Pearson Education Limited. 10.4324/9781351269360

[jar13189-bib-0017] Hu, L. T. , & Bentler, P. M. (1999). Cutoff criteria for fit indexes in covariance structure analysis: Conventional criteria versus new alternatives. Structural Equation Modeling: A Multidisciplinary Journal, 6(1), 1–55.

[jar13189-bib-0018] Jager, K. J. , Tripepi, G. , Chesnaye, N. C. , Dekker, F. W. , Zoccali, C. , & Stel, V. S. (2020). Where to look for the most frequent biases? Nephrology, 25(6), 435–441. 32133725 10.1111/nep.13706 PMC7318122

[jar13189-bib-0019] Jenaro, C. , Verdugo, M. A. , Caballo, C. , Balboni, G. , Lachapelle, Y. , Otrebski, W. , & Schalock, R. L. (2005). Cross‐cultural study of person‐centred quality of life domains and indicators: A replication. Journal of Intellectual Disability Research, 49(Pt 10), 734–739. 10.1111/j.1365-2788.2005.00742.x 16162118

[jar13189-bib-0020] Lavee, E. , & Itzchakov, G. (2021). Good listening: A key element in establishing quality in qualitative research. Qualitative Research, 22(3), 614–631. 10.1177/14687941211039402

[jar13189-bib-0021] Liao, P. , Vajdic, C. , Trollor, J. , & Reppermund, S. (2021). Prevalence and incidence of physical health conditions in people with intellectual disability: A systematic review. PLoS One, 16(8), e0256294. 10.1371/journal.pone.0256294 34428249 PMC8384165

[jar13189-bib-0022] Lobbestael, J. , Leurgans, M. , & Arntz, A. (2011). Inter‐rater reliability of the structured clinical interview for DSM‐IV Axis I disorders (SCID I) and Axis II disorders (SCID II). Clinical Psychology & Psychotherapy, 18(1), 75–79. 10.1002/cpp.693 20309842

[jar13189-bib-0023] Lombardi, M. , Vandenbussche, H. , Claes, C. , Schalock, R. L. , De Maeyer, J. , & Vandevelde, S. (2019). The concept of quality of life as framework for implementing the UNCRPD. Journal of Policy and Practice in Intellectual Disabilities, 16(3), 180–190.

[jar13189-bib-0024] MacCallum, R. C. , Browne, M. W. , & Sugawara, H. M. (1996). Power analysis and determination of sample size for covariance structure modeling. Psychological Methods, 1(2), 130–149.

[jar13189-bib-0025] McCarron, M. , Lombard‐Vance, R. , Murphy, E. , May, P. , Webb, N. , Sheaf, G. , McCallion, P. , Stancliffe, R. , Normand, C. , Smith, V. , & O'Donovan, M. A. (2019). Effect of deinstitutionalisation on quality of life for adults with intellectual disabilities: A systematic review. BMJ Open, 9(4), e025735. 10.1136/bmjopen-2018-025735 PMC6502057 31028039

[jar13189-bib-0026] Merrells, J. , Buchanan, A. , & Waters, R. (2018). The experience of social inclusion for people with intellectual disability within community recreational programs: A systematic review. Journal of Intellectual & Developmental Disability, 43(4), 381–391.

[jar13189-bib-0027] Muthén, L. K. , & Muthén, B. (2017). Mplus user's guide: Statistical analysis with latent variables, user's guide (8th ed.). Muthén & Muthén.

[jar13189-bib-0028] Perry, J. , & Felce, D. (2002). Subjective and objective quality of life assessment: Responsiveness, response bias, and resident:Proxy concordance. Mental Retardation, 40(6), 445–456. 10.1352/0047-6765(2002)040<0445:SAOQOL>2.0.CO;2 12408747

[jar13189-bib-0029] Schaeffer, N. C. , Dykema, J. , & Maynard, D. W. (2010). Interviewers and interviewing. Handbook of Survey Research, 2, 437–471.

[jar13189-bib-0030] Schalock, R. L. , Keith, K. D. , Verdugo, M. Á. , & Gómez, L. E. (2010). Quality of life model development and use in the field of intellectual disability. In Enhancing the quality of life of people with intellectual disabilities (pp. 17–32). Springer.

[jar13189-bib-0031] Schalock, R. L. , Verdugo, M. A. , Jenaro, C. , Wang, M. , Wehmeyer, M. , Jiancheng, X. , & Lachapelle, Y. (2005). Cross‐cultural study of quality of life indicators. American Journal of Mental Retardation, 110(4), 298–311. 10.1352/0895-8017(2005)110[298:CSOQOL]2.0.CO;2 15941366

[jar13189-bib-0032] Sexton, E. , King‐Kallimanis, B. L. , Conroy, R. M. , & Hickey, A. (2013). Psychometric evaluation of the CASP‐19 quality of life scale in an older Irish cohort. Quality of Life Research, 22(9), 2549–2559. 10.1007/s11136-013-0388-7 23504522

[jar13189-bib-0033] Simoes, C. , Santos, S. , & Biscaia, R. (2016). Validation of the Portuguese version of the personal outcomes scale. International Journal of Clinical and Health Psychology, 16(2), 186–200. 10.1016/j.ijchp.2015.11.002 30487862 PMC6225035

[jar13189-bib-0034] Simoes, C. , Santos, S. , & Claes, C. (2015). The Portuguese version of personal outcomes scale: A psychometric validity and reliability study. Intellectual & Developmental Disabilities, 53(2), 129–142. 10.1352/1934-9556-53.2.129 25860451

[jar13189-bib-0035] StataCorp . (2019). Stata statistical software: Release 16. StataCorp LLC.

[jar13189-bib-0036] van Loon, J. , Claes, C. , Vandevelde, S. , Van Hove, G. , & Schalock, R. L. (2010). Assessing individual support needs to enhance personal outcomes. Exceptionality, 18(4), 193–202.

[jar13189-bib-0037] Wang, M. , Schalock, R. L. , Verdugo, M. A. , & Jenaro, C. (2010). Examining the factor structure and hierarchical nature of the quality of life construct. American Journal on Intellectual & Developmental Disabilities, 115(3), 218–233. 10.1352/1944-7558-115.3.218 20441392

[jar13189-bib-0038] Welham, J. , Haire, M. , Mercer, D. , & Stedman, T. (2001). A gap approach to exploring quality of life in mental health. Quality of Life Research, 10(5), 421–429. 10.1023/a:1012549622363 11763204

[jar13189-bib-0039] West, B. T. , & Blom, A. G. (2017). Explaining interviewer effects: A research synthesis. Journal of Survey Statistics and Methodology, 5(2), 175–211.

