# Supplementary material for: Measuring quality of life of adults with intellectual disabilities: Psychometric evaluation of the personal outcomes scale in the United Kingdom
Source: J Appl Res Intellect Disabil. 2024 Jan 2;37(2):e13189. doi: 10.1111/jar.13189 (PMC10952361; doi:10.1111/jar.13189)
Supplement: Supplementary file 1 — Data S1: Supporting Information. [file JAR-37-0-s001.docx]

**Additional Supplementary materials**

**Additional material**

Appendix 1: Item response proportions and % of missing data from use of the Personal Outcome Scale in the UK 2018 - 2022, n = 310

|  | | | | |  |  |
| --- | --- | --- | --- | --- | --- | --- |
| **Item code** | **Item description** | **1 (%)** | **2 (%)** | **3 (%)** | | **Missing (%)** |
| PD1 | Are you able to feed yourself, stand up and sit down, use the toilet, and get dressed by yourself? | 6.77 | 22.9 | 70.32 | | 0 |
| PD2 | Are you able to prepare meals, clean the house, take your medication, and go out by yourself? | 22.26 | 55.81 | 21.91 | | 0 |
| PD3 | Are you learning to do new things? (This might be taking a course, or something that you are doing as part of your development plan) | 40.65 | 42.26 | 16.13 | | 0.97 |
| PD4 | Are you able to demonstrate the skills you have and the things that you can do? | 42.9 | 44.52 | 12.26 | | 0.32 |
| PD5 | Do you have access to the information that you are interested in. For example, being able to find out what’s on TV or what’s happening in your area | 15.81 | 37.42 | 45.81 | | 0.97 |
| PD6 | Do you use a computer, mobile phone, or tablet | 31.29 | 25.81 | 42.58 | | 0.32 |
| SD1 | Do you get given choices about what you want to wear, to eat, and where you would like to go? | 5.16 | 21.61 | 72.9 | | 0.32 |
| SD2 | When you are given choices do you decide for yourself which choice to take? | 3.23 | 29.03 | 67.1 | | 0.65 |
| SD3 | When someone asks you to do something, can you say no? | 5.48 | 29.68 | 64.19 | | 0.65 |
| SD4 | Do people respect your decisions? | 2.9 | 37.1 | 60 | | 0 |
| SD5 | Do you have control over how you spend at least some of your money? | 14.19 | 53.87 | 31.29 | | 0.65 |
| SD6 | Do you get to express your opinion or what you prefer to do, wear, go, and eat | 4.19 | 22.58 | 72.58 | | 0.65 |
| IPR1 | Do you have people you think of as friends (not including paid staff) | 16.45 | 23.87 | 59.03 | | 0.65 |
| IPR2 | How often do you participate in social activities like eating with friends, going to parties, or meeting friends in town? | 21.94 | 50.97 | 26.77 | | 0.32 |
| IPR3 | How often do you communicate or spend time with your family either in person, by phone or email? | 10.97 | 33.55 | 54.52 | | 0.97 |
| IPR4 | How often do you communicate or spend time with your friends either in person, by phone or email? | 23.23 | 35.81 | 40.65 | | 0.32 |
| IPR5 | Do you feel that you are important to your family? | 10 | 10.97 | 76.77 | | 2.26 |
| IPR6 | Do you have a support network - people you can go to for advice and help? | 7.74 | 17.42 | 73.87 | | 0.97 |
| SI1 | Do you talk to people who live or work near to you? | 34.19 | 40 | 25.81 | | 0 |
| SI2 | How many people in the area do you know by name? | 53.55 | 21.94 | 24.19 | | 0.32 |
| SI3 | Do you use shops and services near where you live? (e.g., café, shops, hairdressers, pub, bank, cinema, religious worship, public transport, gym, or sports centre) | 4.52 | 56.13 | 39.03 | | 0.32 |
| SI4 | Do you do things for other people when they need your help? | 29.03 | 41.29 | 28.39 | | 1.29 |
| SI5 | Do people from your community help you with things (including visiting you, taking you places or doing things with you)? | 57.42 | 28.39 | 13.23 | | 0.97 |
| SI6 | Do you go to any local clubs, groups, or community centres near where you live? | 13.55 | 40 | 46.45 | | 0 |
| R1 | Do you have a place in your own home where you can be by yourself? | 3.55 | 7.74 | 88.71 | | 0 |
| R2 | Can you go out of your home and come back in whenever you want? | 26.45 | 16.77 | 56.13 | | 0.65 |
| R3 | Can you have a pet if you want one? | 25.16 | 39.03 | 33.55 | | 2.26 |
| R4 | Can you have a girlfriend or boyfriend if you want? | 10.32 | 12.9 | 76.45 | | 0.32 |
| R5 | Are you allowed to be together as much as you want with your partner/ girlfriend/boyfriend? (Score yes if there is no partner) | 7.1 | 10.97 | 80.32 | | 1.61 |
| R6 | Have you ever voted in recent elections? | 50 | 23.87 | 25.16 | | 0.97 |
| EWB1 | Do you feel safe and secure in your daily environment? | 3.55 | 17.42 | 78.39 | | 0.65 |
| EWB2 | Do you feel successful in the things that you do? | 6.13 | 26.77 | 63.87 | | 3.23 |
| EWB3 | How frequently do you express love, fondness, or affection towards others? | 10 | 37.74 | 50.65 | | 1.61 |
| EWB4 | Are you a happy person? | 4.52 | 25.81 | 69.03 | | 0.65 |
| EWB5 | Are you satisfied with how things are going for you? (In general) | 8.39 | 31.29 | 59.35 | | 0.97 |
| EWB6 | Do you trust the people who are important to you? | 2.58 | 21.61 | 74.84 | | 0.97 |
| PWB1 | In general, how healthy do you feel? | 6.77 | 45.81 | 47.42 | | 0 |
| PWB2 | How often do you exercise, play games, or are active (e.g., fitness, cycling, walking, swimming, or football)? | 25.48 | 42.9 | 31.61 | | 0 |
| PWB3 | Do you get the right amount of rest and relaxation for you? | 8.39 | 19.35 | 71.94 | | 0.32 |
| PWB4 | Is your diet varied and balanced? | 5.16 | 48.06 | 46.77 | | 0 |
| PWB5 | Are you ever in pain or uncomfortable? (Not just today, but generally) | 9.35 | 44.19 | 46.13 | | 0.32 |
| PWB6 | How do you feel when you wake up in the morning? | 13.87 | 32.26 | 53.55 | | 0.32 |
| MWB1 | Do you have enough money to pay for what you really need? (For example, food and bills) | 6.77 | 8.39 | 83.87 | | 0.97 |
| MWB2 | Do you have enough money so you can save some of it? | 11.94 | 24.19 | 62.9 | | 0.97 |
| MWB3 | Do you own things that are important to you? (e.g., your own pictures or photos, TV, mobile phone) | 3.55 | 29.03 | 67.42 | | 0 |
| MWB4 | Do you have a paid job? | 85.81 | 5.48 | 8.06 | | 0.65 |
| MWB5 | Can you lock your door and keep your things safe at home? | 27.1 | 11.29 | 61.61 | | 0 |
| MWB6 | Do you have enough money to make choices (for example what to wear and what to buy?) | 7.42 | 23.23 | 69.03 | | 0.32 |
